# Supplementary material for: Magnetic field compatible circuit quantum electrodynamics with graphene Josephson junctions
Source: Nat Commun. 2018 Nov 5;9:4615. doi: 10.1038/s41467-018-07124-x (PMC6218477; doi:10.1038/s41467-018-07124-x)
Supplement: Supplementary file 1 — Supplementary Information [file 41467_2018_7124_MOESM1_ESM.pdf]

Supplementary Information: Magnetic field compatible circuit quantum electrodynamics with graphene Josephson junctions

J. G. Kroll,[1] W. Uilhoorn,[1] K. L. van der Enden,[1] D. de Jong,[1] K. Watanabe,[2]
T. Taniguchi,[2] S. Goswami,[1] M. C. Cassidy,[1] and L. P. Kouwenhoven[1,3,*]

[1] *QuTech and Kavli Institute for Nanoscience, Delft University of Technology, Delft, 2600 GA, The Netherlands*
[2] *Advanced Materials Laboratory, National Institute for Materials Science, 1-1 Namiki, Tsukuba, 305-0044, Japan*
[3] *Microsoft Station Q Delft, Delft, 2600 GA, The Netherlands*

(Dated: October 12, 2018)

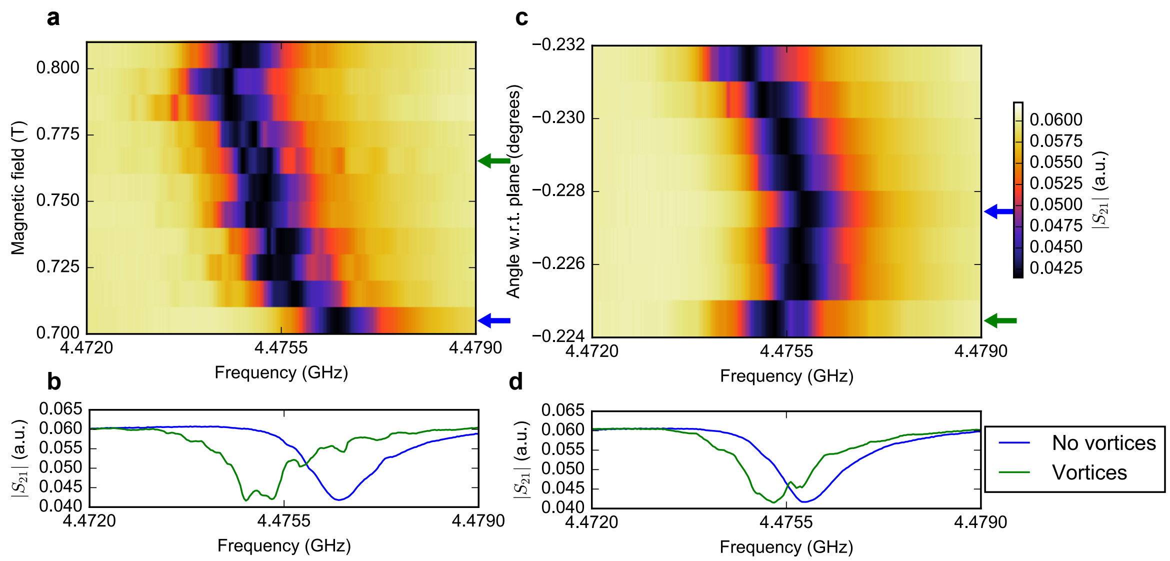

[t!]

Supplementary Figure 1. **Magnetic field alignment. a** Measuring the feedline transmission S_{21} of the NbTiN CPW resonators as a function of probe frequency f as the magnetic field B is increased from 0.7 T to 0.8 T. **b** As B increases the resonator that was previously stable (blue arrow/linecut) is affected by unpinned Abrikosov vortices (green arrow/linecut) giving a reduction in Q_{i} and fluctuations in the resonator frequency on the timescale of the experiment. **c** Varying the angle of the magnetic field with respect to the plane of the superconducting film and measuring the response of the resonator allows the field to be realigned. **d** Although the sweep in magnetic field is hysteretic, the angle is varied until the resonator that was previously affected by vortices (green arrow/linecut) reaches a maximum f_{r} and the fluctautions cease (blue arrow/linecut).

* Leo.Kouwenhoven@Microsoft.com

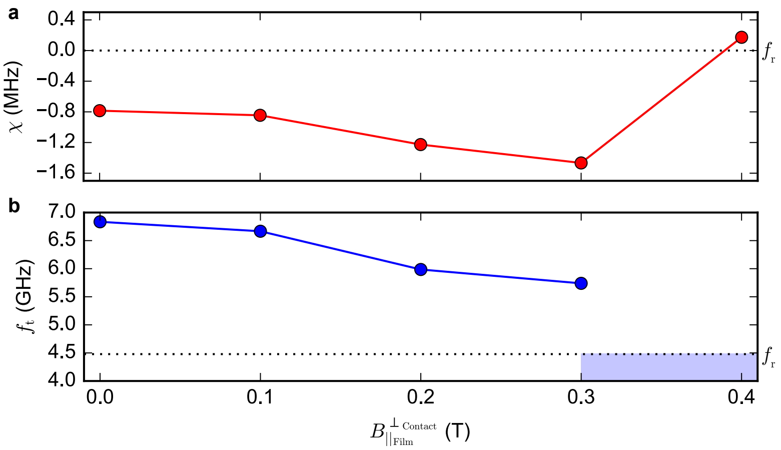

Supplementary Figure 2. **Lead and magnetic field orientation.** **a** Dependence of dispersive shift χ with respect to field $B_{||\mathrm{Film}}^{\perp\mathrm{Contact}}$ applied perpendicular to the length of the junction contacts, but parallel to the film. **b** Extracting f_{t} from χ shows that as $B_{||\mathrm{Film}}^{\perp\mathrm{Contact}}$ is applied the E_{J} reduces significantly. As f_{t} approaches f_{r}, χ increases until f_{t} passes through f_{r} resulting in the sign change of χ. Above $B_{||\mathrm{Film}}^{\perp\mathrm{Contact}} = 0.4\,\mathrm{T}$ the dispersive regime is no longer valid, meaning f_{t} cannot be accurately estimated. These results imply that the E_{J} of the junction is only protected if B is applied along the length of the junction contacts.

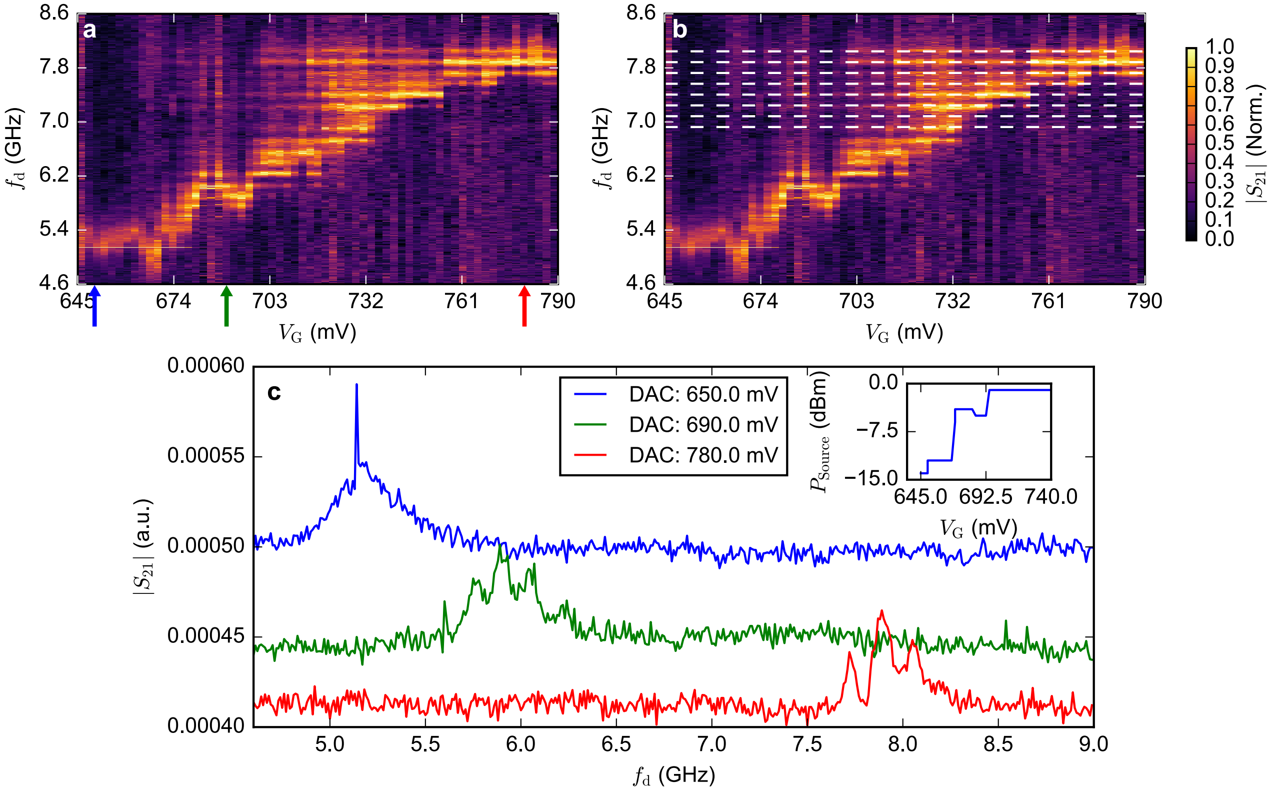

Supplementary Figure 3. **Readout circuit resonance.** **a** Normalised heat map of S_{21} during two-tone spectroscopy as the gate voltage V_G and drive tone f_d is varied. **b** The response of f_t is modulated by background resonances due to a standing wave in the readout circuit caused by an impedance mismatch at the sample, as evidenced by the white lines. **c** Line traces showing $|S_{21}|$ at several V_G values (corresponding to linecuts at coloured arrows in a). The sharp resonance at 5.143 GHz is due to interference effects from an additional $\lambda/4$ resonator multiplexed to the common feedline. The inset demonstrates that above ~ 670 mV the higher power required to drive the transition excites additional modes in the circuit, giving the modulation in response as seen in a. Above 720 mV the resonances become so extreme that it is difficult to analyse the data reliably, so they are excluded from the main text.

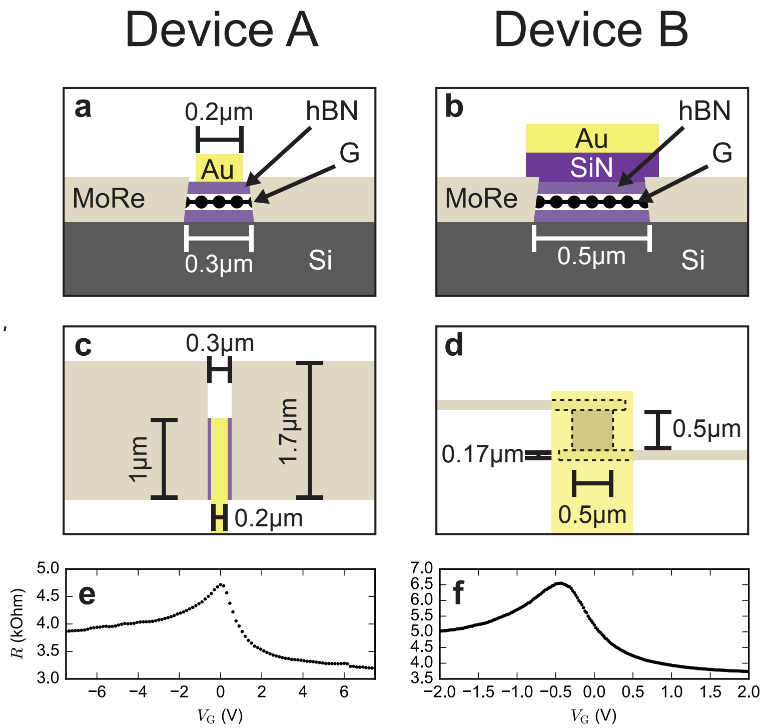

Supplementary Figure 4. **Device fabrication.** **a** Cross sectional diagram showing the geometry of the junction in device A. It is 300 nm in length, with a gate designed to be 200 nm in width. **b** Cross sectional diagram of device B, with the SiN_x/Ti/Au gate giving full coverage of the 500 nm long junction. **c** The junction in device A is 1000 nm wide and contacted by 1700 nm wide MoRe contacts. The 200 nm wide gate can be seen to cover most, but not all of the graphene stack. **d** Device B has a 500 x 500 nm^2 junction that is contacted by thin 170 nm MoRe leads to prevent vortices from forming near the junction. The SiN_x/Ti/Au gate stack is sputtered and designed to give full coverage of the graphene junction. **e** Measurement of the two point resistance R of the contacts and junction for device A at room temperature as the gate voltage V_G is varied. The charge neutrality point can be observed at $V_G = 0$ V. **f** R measurements for device B at room temperature again showing the charge neutrality point, this time offset slightly to $V_G \simeq -0.5$ V. Upon cooling to $T = 15$ mK, the charge neutrality points were observed to shift in both devices.

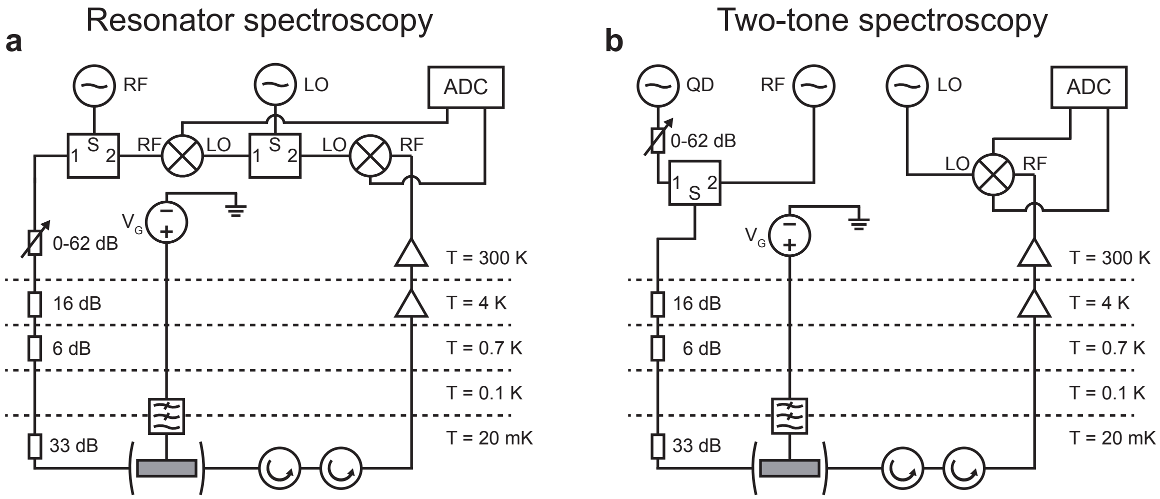

Supplementary Figure 5. **Experimental measurement circuit** All measurements were performed in a dilution refrigerator with a base temperature of 15 mK. The samples were enclosed in a light tight copper box, and thermally anchored to the mixing chamber. The two measurement configurations used for resonator spectroscopy of device A (**a**) and resonator and two-tone spectroscopy of device B (**b**) are pictured. Two coaxial lines and one DC line were used to control the sample. The sample was connected to the DC voltage source by a line that was thermally anchored at each stage and heavily filtered at the mixing chamber by low frequency RC, π and copper powder filters. The line used to drive the feedline input was heavily attenuated to reduce noise and thermal excitation of the cavity, allowing the single photon cavity occupancy to be reached. The output line of the feedline was connected to an isolator (Quinstar QCI-080090XM00) and circulator (Quinstar QCY-060400CM00) in series to shield the sample from thermal radiation from the HEMT amplifier (Low Noise Factory LNF-LNC4-8_C) on the 4K stage.
